# Supplementary material for: Effects of Electrolyte Multivitamins and Neomycin on Immunity and Intestinal Barrier Function in Transported Lambs
Source: Animals (Basel). 2024 Jan 5;14(2):177. doi: 10.3390/ani14020177 (PMC10812564; doi:10.3390/ani14020177)
Supplement: Supplementary file 1 [file animals-14-00177-s001.zip › animals-2764160-supplementary.pdf]

Additional file 1. The original, full- length blots of western blot.

The original, full- length blots of western blot on the jejunal mucosa

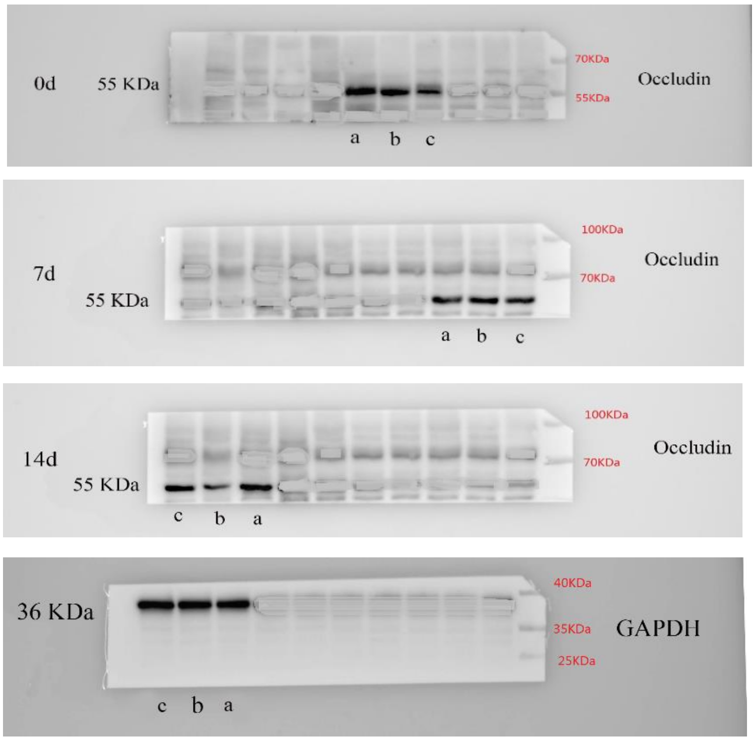

The original, full- length blots of western blot on the colonic mucosa.

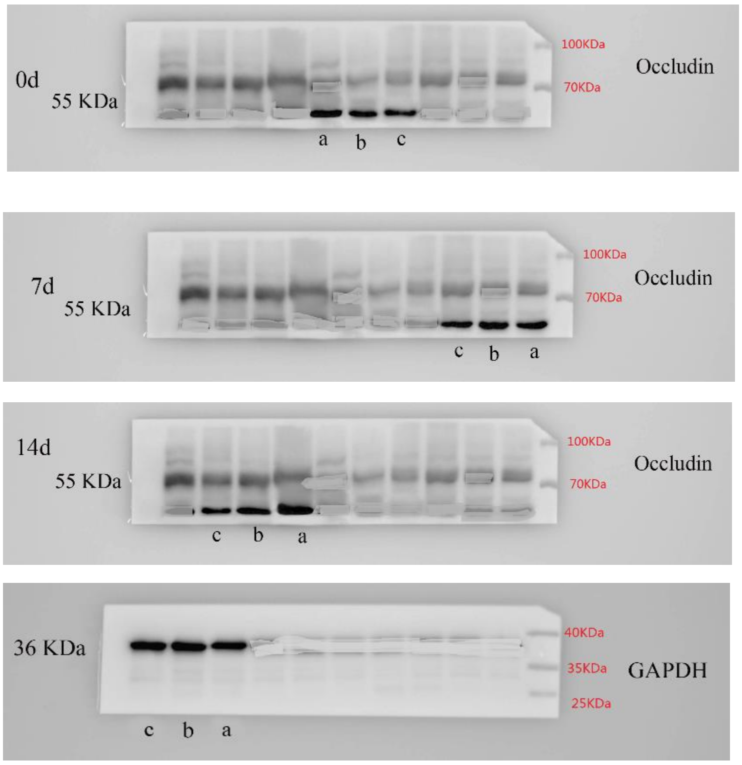

The original, full- length blots of western blot on the jejunal mucosa

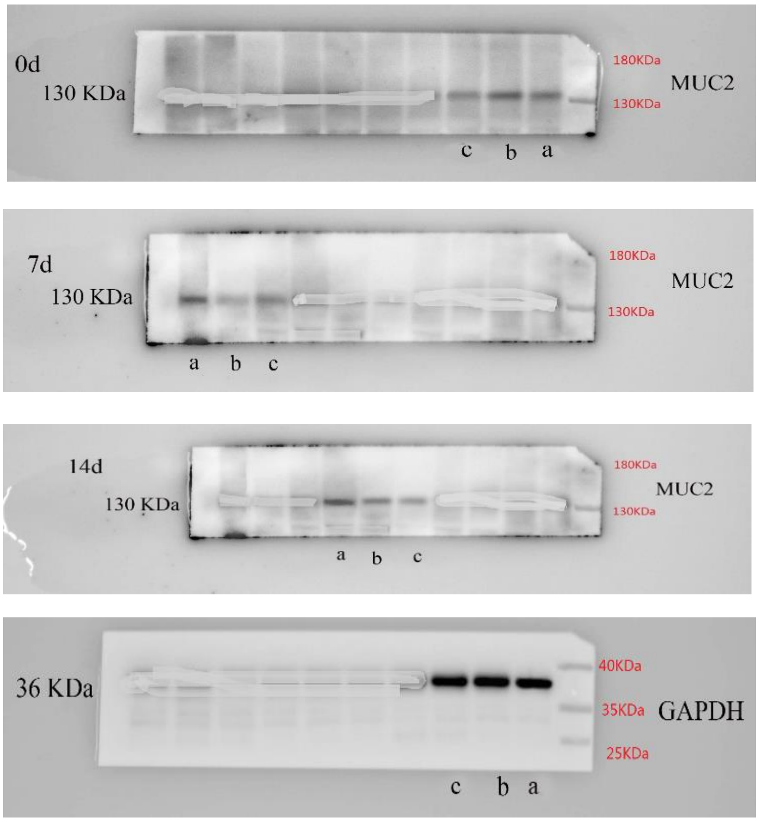

The original, full- length blots of western blot on the colonic mucosa.

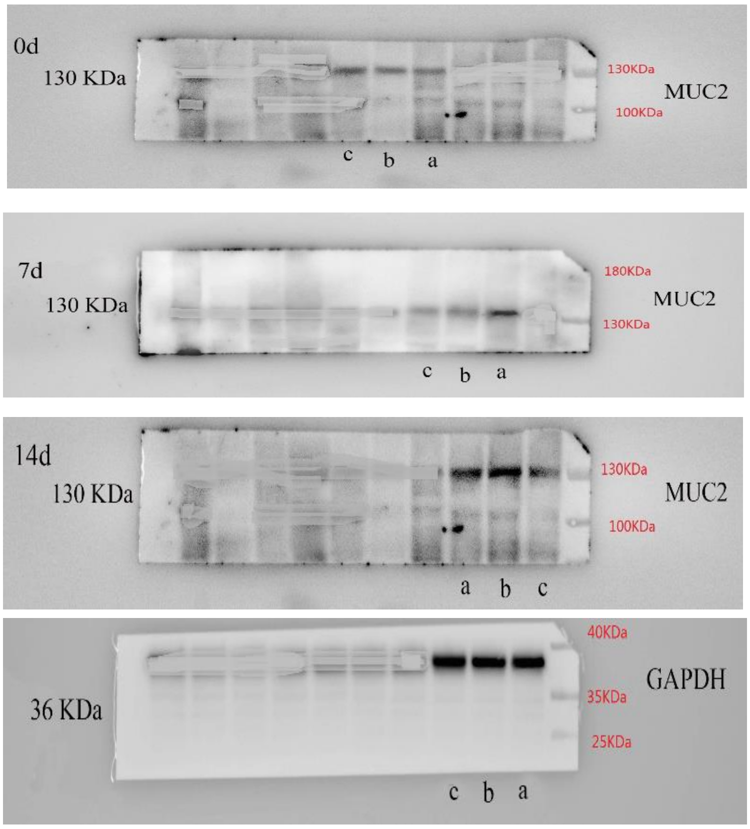

The original, full- length blots of western blot on the jejunal mucosa.

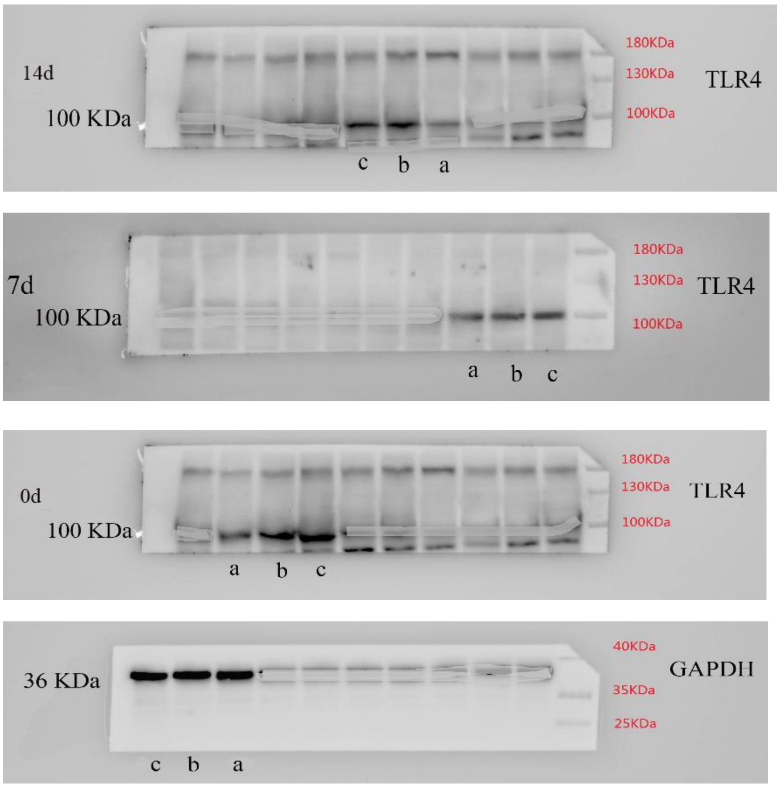

The original, full- length blots of western blot on the colonic mucosa.

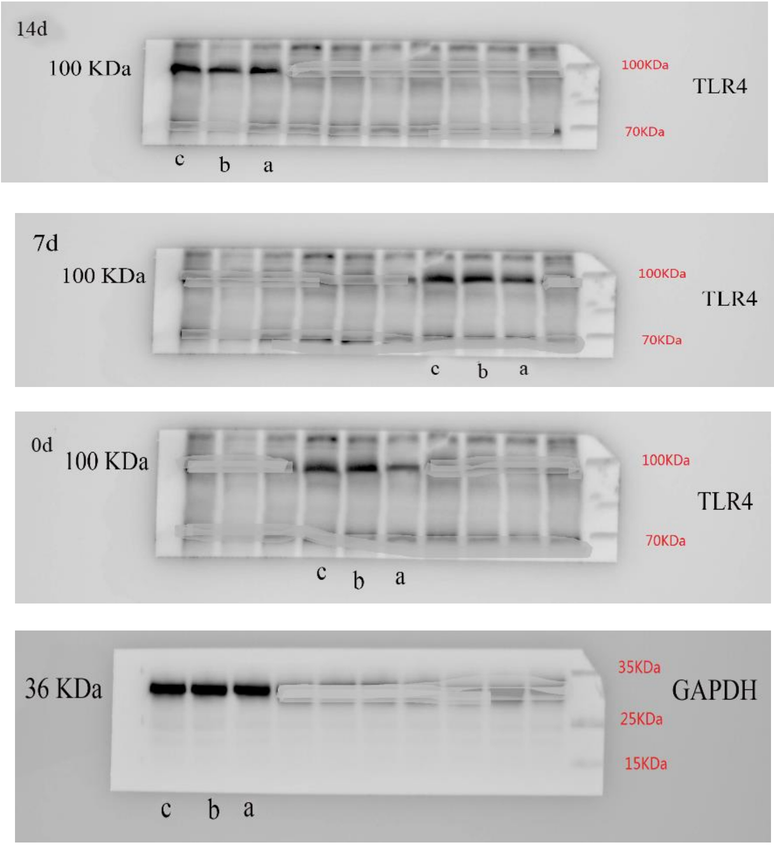

The original, full- length blots of western blot on the jejunal mucosa.

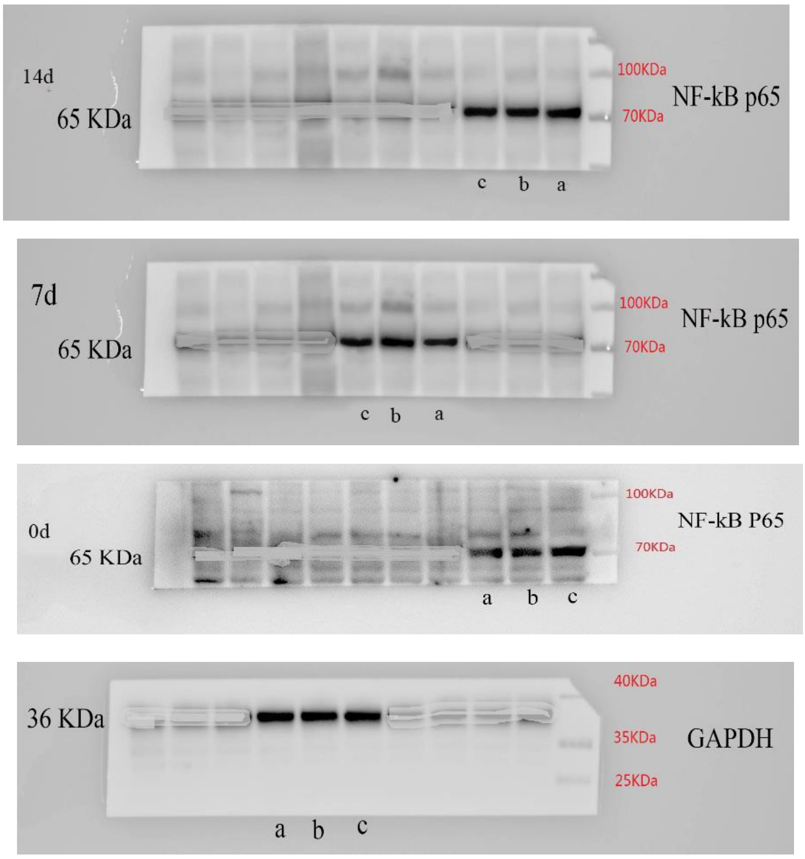

The original, full- length blots of western blot on the colonic mucosa.

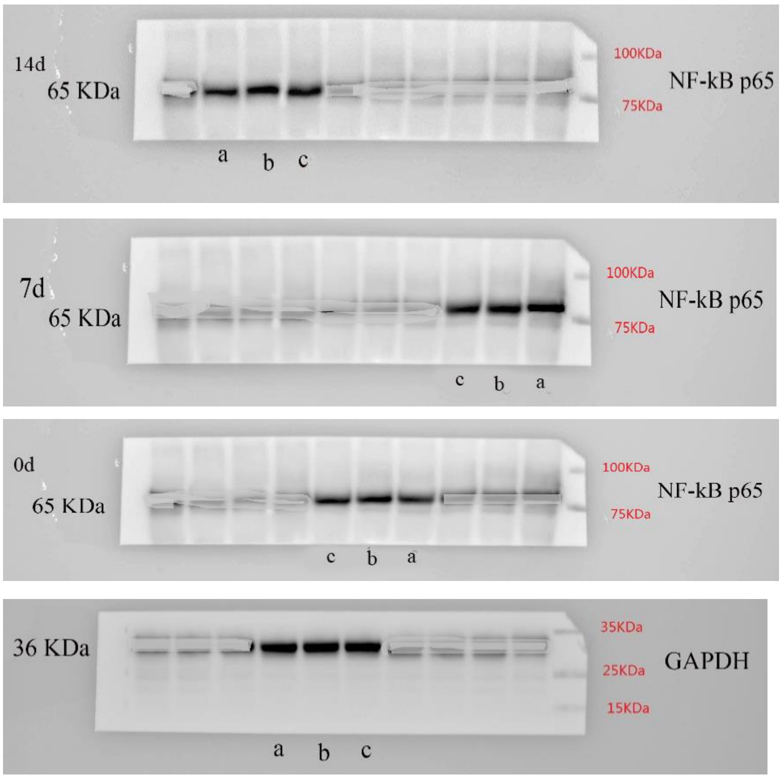

The original, full- length blots of western blot on the jejunal mucosa.

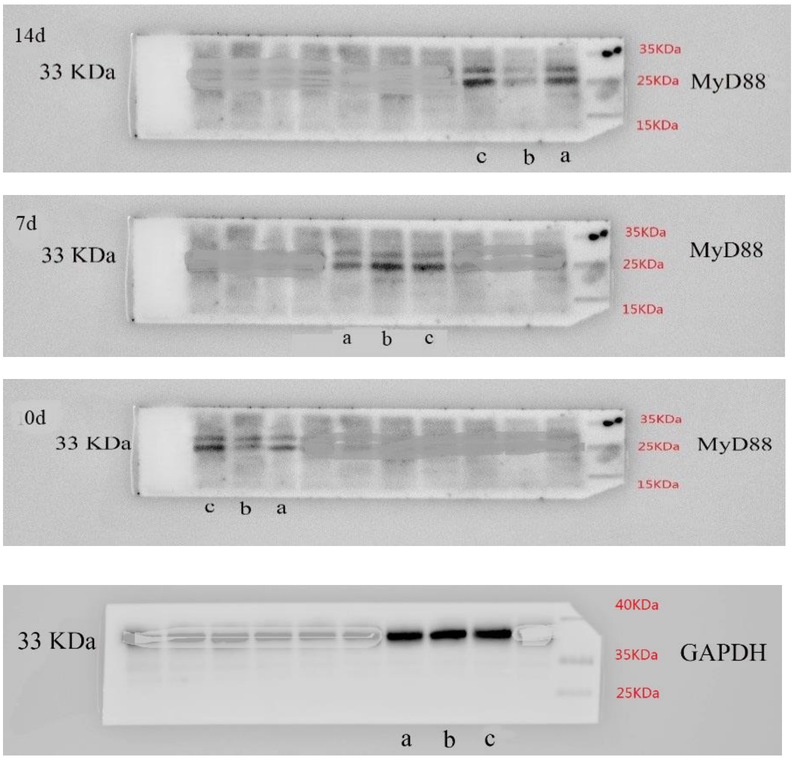

The original, full- length blots of western blot on the colonic mucosa.

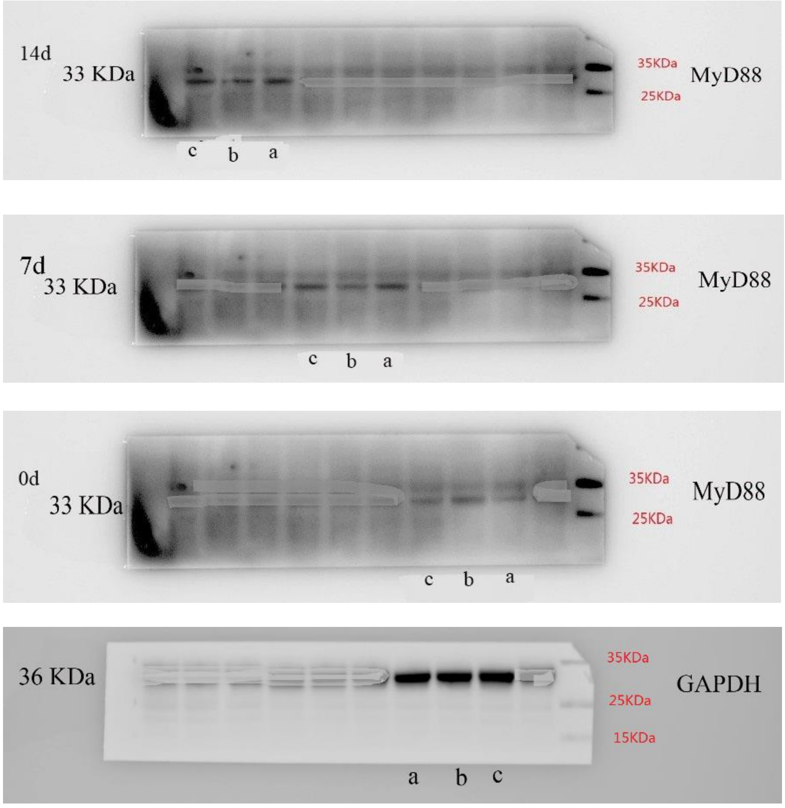

(a) the electrolytic multivitamin group,(b) the neomycin group, (c) the control group.Gel electrophoresis was conducted under the same experimental conditions, and

western blot images were cropped to improve the conciseness of the data.
